# Nutraceutical Extract from Dulse (*Palmaria palmata* L.) Inhibits Primary Human Neutrophil Activation

**DOI:** 10.3390/md17110610

**Published:** 2019-10-25

**Authors:** Maria C Millan-Linares, Maria E Martin, Noelia M Rodriguez, Rocio Toscano, Carmen Claro, Beatriz Bermudez, Justo Pedroche, Francisco Millan, Sergio Montserrat-de la Paz

**Affiliations:** 1 Cell Biology Unit, Instituto de la Grasa, CSIC. Ctra. de Utrera Km. 1, 41013 Seville, Spain; mcmillan@ig.csic.es; 2 Department of Food & Health, Instituto de la Grasa, CSIC. Ctra. de Utrera Km. 1, 41013 Seville, Spain; noe91rm@gmail.com (N.M.R.); mtoscanos@us.es (R.T.); jjavier@cica.es (J.P.); fmillanr@ig.csic.es (F.M.); 3 Department of Cell Biology. Faculty of Biology. Universidad de Sevilla. Av. Reina Mercedes s/n, 41012 Seville, Spain; mariamartin@us.es (M.E.M.); bbermudez@us.es (B.B.); 4 Department of Medical Biochemistry, Molecular Biology, and Immunology. School of Medicine, Universidad de Sevilla, Av. Dr. Fedriani 3, 41071 Seville, Spain; 5 Department of Pharmacology, Pediatrics, and Radiology. School of Medicine, Universidad de Sevilla, Av. Dr. Fedriani 3, 41071 Seville, Spain; cmclaro@us.es

**Keywords:** *Palmaria palmata*, dulse, algae, neutrophils, inflammation, nutraceutic

## Abstract

*Palmaria palmata* L. (Palmariaceae), commonly known as “dulse”, is a red alga that grows on the northern coasts of the Atlantic and Pacific oceans, and is widely used as source of fiber and protein. Dulse is reported to contain anti-inflammatory and antioxidant compounds, albeit no study has investigated these effects in primary human neutrophils. Implication strategies to diminish neutrophil activation have the potential to prevent pathological states. We evaluated the ability of a phenolic dulse extract (DULEXT) to modulate the lipopolysaccharide (LPS)-mediated activation of primary human neutrophils. Intracellular reactive oxygen species (ROS) were measured by fluorescence analysis and nitric oxide (NO) production using the Griess reaction. Inflammatory enzymes and cytokines were detected by ELISA and RT-qPCR. The results show that DULEXT diminished the neutrophil activation related to the down-regulation of TLR4 mRNA expression, deceased gene expression and the LPS-induced release of the chemoattractant mediator IL-8 and the pro-inflammatory cytokines IL-1β, IL-6 and TNF-α. ROS, NO, and myeloperoxidase (MPO) were also depressed. The data indicated that DULEXT has the potential to disrupt the activation of human primary neutrophils and the derived inflammatory and prooxidant conditions, and suggest a new role for *Palmaria palmata* L. in the regulation of the pathogenesis of health disorders in which neutrophils play a key role, including atherosclerosis.

## 1. Introduction

In the western world, macroalgae are used predominantly as a source of functional and technological ingredients in the food, pharmaceutical, and cosmetic industries [1]. Many species of macroalgae have been suggested to have a positive effect on human health, with several key constituents implicated in the prevention of chronic diseases [2]. Most notably, the putative health-promoting properties of seaweed have been attributed to the presence of fiber, bioactive lipids, trace elements, and a diverse range of phenol-based compounds [3]. To date, many authors have investigated these polyphenolic substances with regards to their antioxidant properties and potential use in food applications [4,5,6]. Previous experimental studies with macroalgal extracts or constituents have reported potent anti-inflammatory [7,8] and antioxidant activity [9], albeit there are relatively few studies of these effects on humans [10]. *Palmaria palmata* L. (dulse) belongs to the group of macroalgae known as Rhodophyta or red algae and is native to Europe [11]. Several in vivo animal studies have reported a range of red seaweed species to have anti-inflammatory activity [12,13,14,15]. Specifically, for *P. palmata*, this anti-inflammatory activity has been attributed to its relatively high proportion of eicosapentaenoic acid (EPA) concentration and its favorably low ω-6/ω-3 ratio (0.13) [16,17,18,19]. Numerous studies have shown that methanol, ethanol, and aqueous extracts of *P. palmata* exhibit antioxidant activity [20,21]. Potential health benefits derived from the consumption of dulse have received a great deal of attention [22,23]. Nevertheless, so far there is a relatively small body of literature concerning both anti-inflammatory and antioxidant properties of *P. palmata* compounds in humans [7].

Inflammation is a physiopathological phenomenon involved in numerous diseases such as cardiovascular diseases and cancer [24]. Neutrophils represent the most abundant pool of leukocytes in the human blood and play a crucial role in inflammation, standing as the first line of defense of the innate immune system [25]. The primary function of the neutrophils is to recognize, phagocytose, and kill invading microorganisms, and this killing is achieved via the release of proteolytic enzymes, pro-inflammatory cytokines and the generation of reactive oxygen species (ROS) into the phagosome [26,27]. Following this killing, neutrophil apoptosis is required for the effective resolution of inflammation, and defects in the regulation of this process are implicated in the pathogenesis of numerous disorders [28,29,30,31,32].

Therefore, the main aim of this study was evaluating the ability of the dulse (*Palmaria palmata* L.) extract (DULEXT) to modulate the lipopolysaccharide (LPS)-mediated activation on primary human neutrophils, which are involved in the pathogenesis of numerous disorders.

## 2. Results

### 2.1. DULEXT Impairs Oxidant Conditions

**Figure 1:**
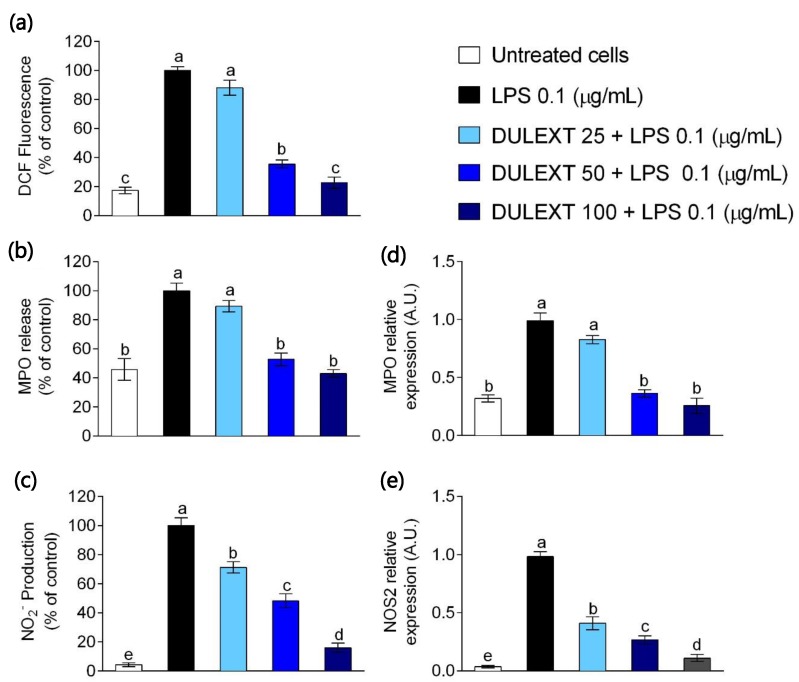
Intracellular reactive oxygen species (ROS) (**a**), myeloperoxidase (MPO) (**b**), and nitrite (**c**) production, expressed as percentage of fluorescence/absorbance, and MPO (**d**) and NOS2 (**e**) mRNA levels relative to cells treated with lipopolysaccharide (LPS) (0.1 μg/mL), after the treatment of neutrophils with dulse (*Palmaria palmata* L.) extract (DULEXT), at 25, 50, and 100 μg/mL for 6 h. Values are presented as means ± SD (*n* = 3) and those marked with different letters were significantly different (*P* < 0.05).

The antioxidant capacity of DULEXT is a potential bioactive property. For that reason, we evaluated the preventive effects of several doses of DULEXT on the oxidative state. Considering LPS-activated human neutrophils as a control of 100% intracellular ROS production, the extract induced a dose-dependent decrease (Figure 1a). Similar effects were observed after quantifying myeloperoxidase (MPO) release in culture supernatants compared to LPS-stimulated cells, depressing pro-oxidant enzyme concentration in the presence of DULEXT (Figure 1b). There was also a remarkable decrease at a dose of 50 µg/mL. In line with these effects, MPO gene expression was down-regulated by DULEXT (Figure 1d). Nitrite (NO_2_^−^) production, as an indicator of nitric oxide (NO) generation, also refers to oxidative conditions. When measuring the production of intracellular nitrites compared to LPS-treated neutrophils, DULEXT reduced its release to the culture medium (Figure 1c). Quantification showed the extract induced a dose-dependent decrease. Relative Nos2 mRNA expression levels detected by RT-qPCR confirmed this potential antioxidant effect (Figure 1e). In this case, at 25 µg/mL, the decreasing trend was remarkable.

### 2.2. DULEXT Down-Regulates TRL4 and Pro-Inflammatory Cytokine Gene Expression 

**Figure 2:**
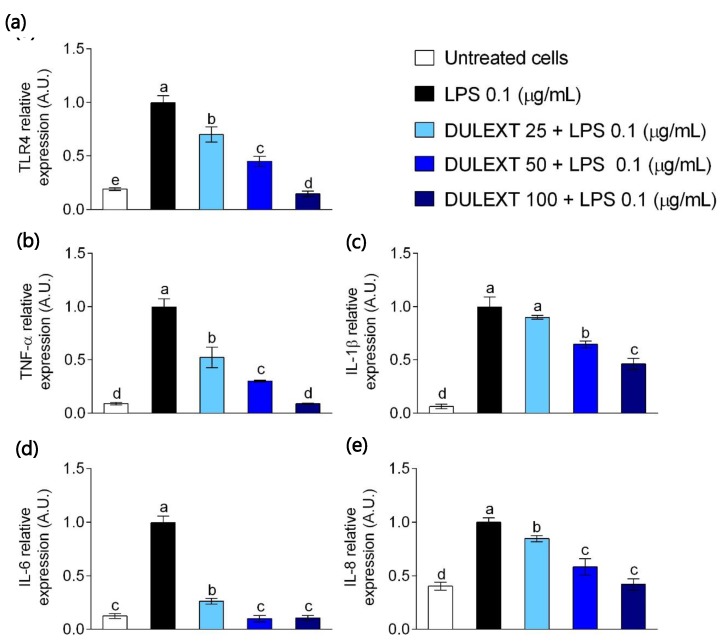
Gene expression of TLR4 (**a**), TNF-α (**b**), IL-1β (**c**), IL-6 (**d**), and IL-8 (**e**) relative to cells treated with LPS (0.1 μg/mL) after the treatment of neutrophils with DULEXT at 25, 50, and 100 μg/mL for 6 h. Values are presented as means ± SD (*n* = 3) and those marked with different letters were significantly different (*P* < 0.05).

The anti-inflammatory effects of *Palmaria palmata* L. compounds have not been previously tested in primary human neutrophils. LPS recognition by TLR4 leads to the release of pro-inflammatory cytokines, including TNF-α, IL-β, and IL-6. The transcriptional activity of those genes was tested in LPS-activated human neutrophils. TLR4 mRNA levels diminished in LPS-treated cells in the presence of DULEXT in a dose-dependent manner (Figure 2a). We found a significant reduction after treatment with 100 µg/mL of DULEXT. Similar effects were observed when measuring TNF-α, IL-β, and IL-6 gene expression (Figure 2b–d, respectively). From these graphs we concluded that the pro-inflammatory cytokines diminished their release, but not in the same way. TNF-α value was reduced by almost half with the lowest 25 µg/ml dose, whereas IL-β showed quite a progressive decrease and IL-6 expression was dramatically reduced from the initial treatment. DULEXT also down-regulated the relative expression of LPS-induced release of IL-8, a chemoattractant mediator (Figure 2e). The results showed a continuous decrease in a dose-dependent manner from 25 to 50 and 100 µg/mL DLUEXT. 

### 2.3. DULEXT Reduces the Release of Pro-Inflammatory Cytokines

**Figure 3:**
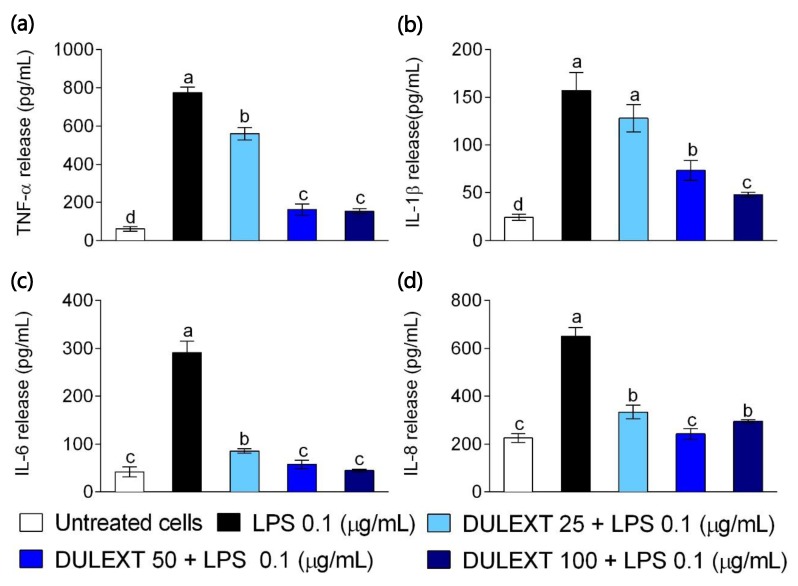
Secretion of pro-inflammatory TNF-α (**a**), IL-1β (**b**), IL-6 (**c**), and IL-8 (**d**) in cells treated with LPS (0.1 μg/mL) after the treatment of neutrophils with DULEXT at 25, 50, and 100 μg/mL for 6 h. Values are presented as means ± SD (*n* = 3) and those marked with different letters were significantly different (*P* < 0.05).

Inflammatory conditions were also tested by the quantification of cytokines in culture supernatants. Similar results for TNF-α, IL-β, IL-6, and IL-8 were obtained after stimulating primary human neutrophils in the presence of LPS (control samples) with 25, 50, and 100 µg/mL of DULEXT. The treatment reduced the secretion of the pro-inflammatory cytokines in all cases. In Figure 3a there was a trend toward decreasing TNF-α release, which became less than half of the initial 50 µg/mL concentration. The quantification of IL-β cytokine revealed that there was a gradual decline according to the extract dose (Figure 3b). Figure 3c shows the dramatic decline by as much as a third of the IL-6 concentration from the first 25 µg/mL DULEXT treatment. From the data in Figure 3d, there was no significant difference when comparing the release of the chemoattract mediator IL-8 after treatments, though the reduction with only 25 µg/mL dulse extract was remarkable.

### 2.4. DULEXT Down-Regulates Neutrophil Elastase Gene Expression and Impairs its Enzymatic Activity

**Figure 4:**
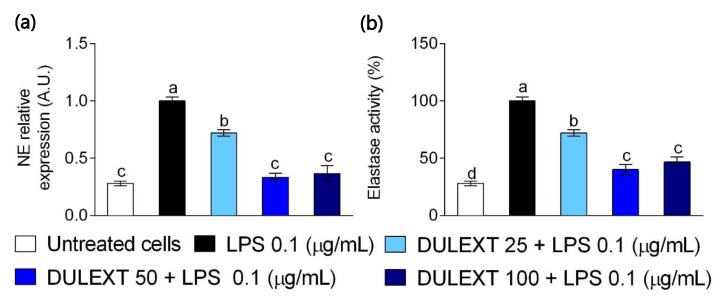
Neutrophil elastase mRNA levels (**a**) and activity (**b**) relative to cells treated with LPS (0.1 μg/mL) after the treatment of neutrophils with DULEXT at 25, 50, and 100 μg/mL for 6 h. Values are presented as means ± SD (*n* = 3) and those marked with different letters were significantly different (*P* < 0.05).

The following inflammatory event to be evaluated was the gene expression (Figure 4a) and release (Figure 4b) of neutrophil elastase. The presence of DULEXT down-regulated and significantly inhibited the mRNA levels and release of neutrophil elastase induced by LPS for all tested concentrations. The inhibition of this event may have had a suppressive effect on inflammation, the activation of neutrophils, and the capture/elimination of pathogens by decreasing the inflammatory stimulus that came from the genetic material released.

### 2.5. Effect of DULEXT on Neutrophil Subsets

**Figure 5:**
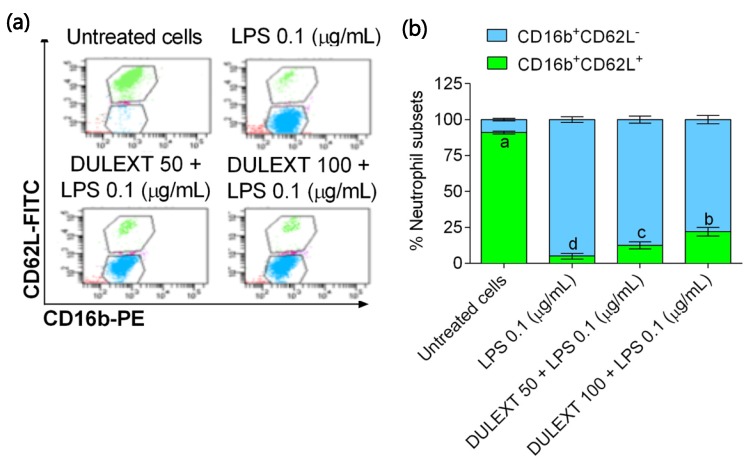
Fluorescence activated cell sorting (FACS) analysis (**a**) of neutrophil surface markers CD16 and CD62L after 6 h incubation with or without LPS (0.1 μg/mL) after the treatment of neutrophils with DULEXT at 25, 50, and 100 μg/mL. CD16^+^CD62L^−^ activated neutrophils (blue color) and CD16^+^CD62L^+^ non-activated neutrophils (green color) (**b**). Values are presented as means ± SD (*n* = 3) and those marked with different letters were significantly different (*P* < 0.05).

Herein, the degree of activation in human neutrophils was determined by measuring membrane markers with flow cytometry after LPS incubation in the presence or absence of DULEXT. The upper panel of Figure 5 (Figure 5a) depicts a flow cytometer dot plot, showing the uniform expression of CD16 and CD62L by primary human neutrophils. The control pool of neutrophils consisted of a clearly marked phenotype with 95% of CD16^+^CD62L^+^ (Figure 5b). The CD16^+^CD62L^+^ cells were almost depleted after LPS administration (–98%, *p* < 0.001). At this time point, banded (CD16^+^CD62L^+^) neutrophils slightly appeared after LPS-DULEXT co-incubation, and these populations raised 10–15% of the total neutrophils (*p* < 0.05). DULEXT administration seemed to return neutrophil phenotype to basal conditions.

## 3. Discussion

Traditionally, algae have been widely used as a food source in Asian countries but, in recent years, they have become an interesting source of industrial products, including bioactive compounds. Their composition includes not only proteins, lipids, fiber, vitamins, and minerals, but also nutraceutical substances such as phenols [18,33].

The interest in red algae as a functional food product has increased due to their anti-inflammatory and anti-oxidant activity [34,35]. In the present work, a phenolic extract of *Palmaria palmata* L. (dulse) was used for the first time in order to test its ability to impair primary human neutrophils activated by LPS and its potential for regulating pathological states.

Several experiments were carried out to determine the antioxidant capacity of dulse extract (DULEXT). Parameters such as intracellular ROS production, NO, or MPO generation and expression enabled the analysis of oxidative stress conditions. The extract suppressed both the intracellular production of ROS and the MPO release in culture supernatants, as well as MPO gene expression, compared to control conditions induced by LPS in human neutrophils. Similar effects were observed for nitrite production and mRNA Nos2 expression. In all cases, the tendency was dose-dependent. Few studies have been focused on the relationship between phenol compounds and the antioxidant capacity of algae [36,37]. Yuan et al. (2005) [36] tested the antioxidant and antiproliferative effects of dulse extracts, and Machu et al. (2015) [18] studied phenolic compounds and activity in some commercial food products, including Palmaria. However, this study is the first to demonstrate the antioxidant capacity of the dulse phenolic extract. 

Several reports have shown that some seaweeds have anti-inflammatory activity [7,8], although very little was found in the literature regarding experiments with dulse extracts or compounds in animal cells [1,38]. In this context, Robertson et al. (2015) [1] tested the anti-inflammatory potential of several lipid algal extracts, including Palmaria, in human macrophages. Lee et al. (2017) [38] demonstrated anti-inflammatory effects in murine macrophages when testing certain Palmaria compounds. Our study supports evidence of the anti-inflammatory properties of *Palmaria palmata* L. in primary human neutrophils. On the one hand, DULEXT decreased TLR4 mRNA levels in LPS-stimulated cells in a dose-dependent way and, as could be expected, decreased the gene expression of pro-inflammatory cytokines, such as TNF-α, IL-1β, and IL-6 and the chemoattractant IL-8. Recognition of LPS mainly by TLR4 initiates several signaling cascades, leading to the activation of NF-κB and MAPK pathways that mediate the expression of inflammatory cytokines and transactivation of pro-inflammatory enzymes such as iNOS and COX-2 in cells of innate system [39,40]. All of these effects likely contribute to the idea of DULEXT as a disruptor of TLR-mediated pro-inflammatory responses in innate system. On the other hand, DULEXT reduced the levels of the mentioned cytokines released to the cell culture supernatants. However, contrary to expectations, the DULEXT doses did not interfere equally with the analyzed gene expressions or the quantification of the inflammatory mediators. IL-6 dramatically reduced its transcriptional activity and release to the medium with the lowest dose, suggesting an original role in the inflammation processes. Future studies also need to be undertaken in order to explain the effects of decreasing levels of IL-8 in the culture supernatants, due to the fact that it did not occur as gradually as expected.

It is now well established from a variety of studies that there are many molecules involved in the inflammatory processes. Among the proteins described in neutrophil granules, there are neutrophil serine proteases, which include human neutrophil elastase (NE). Nauseef et al. (2016) [30] describes these serine proteinases as crucial for antimicrobial action and tissue degradation. Kettritz et al. (2016) [41] highlights other processes in which they are involved, including autoimmmunity, metabolic processes, and cancer [30,41]. The present study demonstrates for the first time that in human activated neutrophils, DULEXT down-regulates NE mRNA expression and diminishes the release of the enzyme. These results are similar to those obtained in our experiments when testing inflammatory mediators such as cytokines, and once again we conclude that the mechanism is dose-dependent. Further experiments are needed due to the fact that this proteinase has been scarcely tested, albeit there are many forms of evidence of its activity in pathological states [42].

Nauseef et al. (2016) [30] refers to inflammatory states describing several processes including neutrophil activation, which leads to neutrophil migration into tissues and the production of cytokines. Only human neutrophils express CD16b, whose structure is altered after neutrophil activation. CD62L also plays an important role when neutrophils move to the damaged area [40]. With respect to the degree of activation of human neutrophils, we designed flow cytometry experiments in order to measure those membrane markers. Dot plots showed very little difference after LPS activation and treatment with two doses of DULEXT in CD16b and CD62L expressions. Furthermore, the quantification of neutrophil subsets indicated that the extract may slightly recover cell phenotypes. These results may become experimental evidence of the management of inflammation states, both preventing chronic outcome and repairing damage. Nevertheless, our study is the first to show this balance in human neutrophils.

## 4. Materials and Methods

### 4.1. Preparation of Dulse (Palmaria palmata L.) Extract (DULEXT)

Red (*Palmaria palmata* L.) algae were analyzed in the present study. Fresh samples, supplied by the factory Algamar (Redondela, Pontevedra, Spain), were freeze-dried for 24 h before analysis. Two hundred and fifty grams of freeze-dried *Palmaria palmata* L. were extracted with methanol overnight at room temperature, filtered, and concentrated by rotary evaporation at 40 °C, as previously described [36]. The concentrated extract was further washed with hexane and the lower methanol phase was then extracted with H_2_O + ethyl acetate. The lower H_2_O–methanol layer was then extracted with 1-butanol and the upper butanol layer was concentrated by rotary evaporation to obtain a light brown residual powder. The dulse extract (DULEXT) was solubilized in 0.1% ethanol for use in assays.

Both quantitative and qualitative analyses of phenolic compounds were carried out according to COI/T20/29 with some modifications. This protocol is based on the direct extraction and quantification of the phenolic minor polar compounds using a methanol solution and HPLC [43]. Once extracted, an aliquot of the supernatant phase was taken, filtered, and injected into a HPLC system that included a C18 reverse-phase column (25 cm × 4.6 mm), type Spherisorb ODS-2 (5 mm), and a spectrophotometric UV detector at 280 nm. Phenol content was identified and quantified after the measurement of the areas of the related chromatographic peaks and expressed in microgram per milligram of dry weight [44]. Table 1 includes detailed data concerning the composition of the isolated DULEXT.

### 4.2. Blood Collection and Neutrophil Isolation

This study was conducted according to the guidelines of good clinical practice. Peripheral venous blood was isolated from healthy adult volunteers (<35 years old) from the University Hospital Virgen del Rocio in Seville. The investigation conformed to the principles outlined in the Helsinki Declaration of the World Medical Association. This study has the agreement of the Human Clinical Commission and Ethics Committee at the UHVM (PI-0008-2017). The donors were recognized as healthy, according to medical history and routine laboratory tests. The donors declared that they were non-smokers and were not taking any medication. Blood samples were immediately collected into K3EDTA-containing vacutainer tubes (Becton Dickinson, NJ, USA). Neutrophils were isolated by dextran sedimentation in a Ficoll Histopaque gradient (Sigma-Aldrich Chem., St. Louis, MO, USA), and erythrocytes were removed by hypotonic lysis. The purity of neutrophil preparation was >97% by trypan blue exclusion. Following isolation, the cells were suspended in an RPMI 1640 medium supplemented with L-glutamine, penicillin, streptomycin, and 1% heat-inactivated fetal bovine serum. To antioxidant and anti-inflammatory assays, the neutrophils were seeded at a density of 3 × 10^6^ cells/mL. The cells were treated with 0.1 µg/mL LPS from *Escherichia coli* 055:B5 (Sigma-Aldrich, St Louis, MO, USA) in the presence or absence of DULEXT (25, 50, and 100 µg/mL) for 6 h.

### 4.3. Cytotoxicity Assay

Neutrophils seeded in 96-well plates (1 × 10^6^ cells/well) were incubated in the presence or absence of different DULEXT concentrations (up to 200 µg/mL) for 6 h. At the end of the exposure time, the effect on cell growth/viability was analyzed by a MTT colorimetric assay [45]. Cell survival was measured as the percentage of absorbance compared to that obtained in control cells (non-treated cells).

### 4.4. Intracellular Reactive Oxygen Species (ROS)

Intracellular ROS production was measured using 2’-7’-dichloro-dihydro-fluorescein diacetate (DCFH-DA, Sigma-Aldrich). DCFH-DA enters cells and is hydrolyzed by intracellular esterases to form the non-permeable and non-fluorescent 2’-7’-dichloro-dihydro-fluorescein (DCFH), which is rapidly oxidized in the presence of ROS to the highly fluorescent 2’-7’-dichloro-fluorescein (DCF). Fluorescence intensity was measured as previously described [46]. The results were expressed as percentage of intracellular ROS production and were compared with those obtained upon treatment of cells with LPS (0.1 µg/mL), as a positive control (100% ROS production).

### 4.5. Nitrite Production

Nitrite production, as an index of nitric oxide (NO) generation, was determined by the Griess reagent (Sigma-Aldrich). The culture supernatant (100 μL) was transferred to a 96-well plate and then mixed with 100 μL of Griess reagent [47]. The absorbance was measured at a wavelength of 540 nm in a BioTek plate reader. To calculate the concentration, a standard curve with sodium nitrite was used.

### 4.6. Cytokine Release. 

In culture supernatants, IL-1β, IL-6, IL-8, TNF-α, and MPO concentrations were determined using commercial ELISA kits (eBioscience). The values were expressed as picogram per milliliter and calculated from the standard curves for each test. Absorbance was measured at 450 nm on a Multiskan Spectrum plate reader.

### 4.7. RNA Isolation and Real-Time Quantitative PCR Analysis. 

Total RNA was extracted by using Trisure Reagent (Bioline). RNA quality was assessed by the A_260_/A_280_ ratio in a NanoDrop ND-1000 Spectrophotometer (Thermo Scientific). RNA (250 ng) was subjected to reverse transcription (iScript, BioRad). An amount of 20 ng of the resulting cDNA was used as a template for real-time PCR amplifications. The mRNA levels for specific genes were determined in a CFX96 system (BioRad). For each PCR reaction, a cDNA template was added to a Brilliant SYBR green QPCR Supermix (BioRad) containing the primer pairs for the corresponding gene. Glyceraldehyde 3-phosphate dehydrogenase (GAPDH) and hypoxanthine phosphoribosyl transferase (HPRT) were used as housekeeping genes. The sequence and information for the primers are shown in Cardeno et al. (2015) [44]. All amplification reactions were performed in triplicate and average threshold cycle (Ct) numbers of the triplicates were used to calculate the relative mRNA expression of candidate genes. The magnitude of change of mRNA expression for candidate genes was calculated by using the standard 2^−(ΔΔCt)^ method. All data were normalized to the content of housekeeping genes and expressed as percentage of control. 

### 4.8. Human Neutrophil Elastase Inhibition Assay

The inhibition assay was performed, as described previously [48]. Briefly, a buffer solution containing 200 mM Tris-HCl, pH 7.5, 0.01% bovine serum albumin, 0.05% Tween-20, and 20 mU/mL of human neutrophil elastase was added to black, flat-bottom 96-well microtiter plate culture cell supernatant. Reactions were initiated by the addition of 25 µM elastase substrate N-methylsuccinyl-Ala-Ala-Pro-Val-7-amino-4-methylcoumarin in a final reaction volume of 100 µL/well for 30 min. Color reactions were obtained on a Fluoroskan Ascent FL fluorescence microplate reader (Thermo Electron, MA) with excitation and emission wavelengths at 355 and 460 nm, respectively. Several concentrations of purified elastase enzyme from human neutrophils (EMD Chemicals Inc., Billerica, MA, USA) were used as standards.

### 4.9. Flow Cytometry

Membrane expression of CD16 (PE anti-human CD16) and CD62L (FITC anti-human CD62L) on neutrophils was assessed by flow cytometry. According to the manufacturer’s instructions, 106 of the purified neutrophils after in vitro stimulation with or without LPS were incubated with antibodies at room temperature, in the dark, for 15 minutes, followed by fixation and lysing of erythrocytes with 20× volume of fluorescence activated cell sorting (FACS) lysing solution (BD Bioscience, San Jose, CA, USA). Fluorescence intensity was measured by FACS Canto II (Becton Dickinson Immunocytometry Systems, Mountain View, CA, USA) and calibrated using FACS Canto II cell analyzer software (Becton Dickinson). Mean fluorescence intensity (MFI) of 10^4^ counted cells was measured in each sample. Neutrophils were gated as forward scatter high (FSC^high^)-side scatter high (SSC^high^) and CD16^high^ cells. Expression levels were presented as MFI corrected for the non-specific binding of isotype control antibodies on neutrophils from the same donor.

### 4.10. Statistical Evaluation. 

All values in the figures and text are expressed as arithmetic means ± standard deviation (SD). Experiments were carried out in triplicate. Data were evaluated with Graph Pad Prism Version 5.01 software. The statistical significance of any difference in each parameter among the groups was evaluated by one-way analysis of variance (ANOVA), using Tukey’s multiple comparisons test as a post-hoc test. *P*-values of < 0.05 were considered statistically significant.

## 5. Conclusion

In conclusion, the results of our study suggest that the dulse extract (DULEXT) from *Palmaria palmata* L. plays an important role in neutrophil activation contributing to the attenuation of proinflammatory and prooxidant episodes that could be considered as a new strategy for chronic inflammatory diseases, suggesting novel benefits derived from its consumption as nutraceutical in many diseases.

## Figures and Tables

**Table 1:** Total phenolic content in DULEXT.

Polyphenol	Chemical Structure	Concentration μg/mg of Extract
4-hydroxybenzoic acid (HBA)	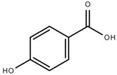	6.3 ± 0.7
Epicatechin (EC)	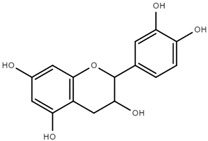	3.6 ± 0.5
Epigallocatechin (ECG)	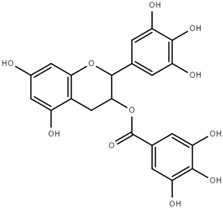	2.8 ± 0.5
Gallic acid (GA)	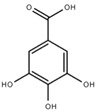	1.6 ± 0.2
Epigallocatechin gallate (ECGg)	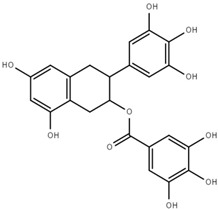	1.0 ± 0.2
Catechin (C)	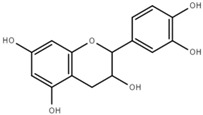	0.7 ± 0.1
Catechin gallate (CG)	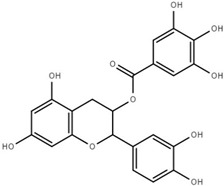	0.2 ± 0.1
Total phenolic content		18.9 ± 2.7
